# Medication use in juvenile uveitis patients enrolled in the Childhood Arthritis and Rheumatology Research Alliance Registry

**DOI:** 10.1186/s12969-016-0069-5

**Published:** 2016-02-16

**Authors:** Lauren A. Henderson, David Zurakowski, Sheila T. Angeles-Han, Andrew Lasky, C. Egla Rabinovich, Mindy S. Lo, for the CARRA Registry Investigators

**Affiliations:** Division of Immunology, Boston Children’s Hospital, Boston, MA and Department of Pediatrics, Harvard Medical School, 1 Blackfan Circle, Karp Building, 10th Floor, Boston, MA 02115 USA; Departments of Anesthesia and Surgery, Boston Children’s Hospital and Harvard Medical School, Boston, MA USA; Department of Pediatrics and Ophthalmology, Emory University School of Medicine, Atlanta, GA USA; Department of Pediatric Rheumatology, Randall Children’s Hospital at Legacy Emanuel, Portland, OR USA; Division of Rheumatology, Department of Pediatrics, Duke Children’s, Durham, NC USA

**Keywords:** Uveitis, Juvenile idiopathic arthritis (JIA), Idiopathic uveitis, DMARD, Infliximab, Adalimumab, Methotrexate

## Abstract

**Background:**

There is not yet a commonly accepted, standardized approach in the treatment of juvenile idiopathic uveitis when initial steroid therapy is insufficient. We sought to assess current practice patterns within a large cohort of children with juvenile uveitis.

**Methods:**

This is a cross-sectional cohort study of patients with uveitis enrolled in the Childhood Arthritis and Rheumatology Research Alliance (CARRAnet) registry. Clinical information including, demographic information, presenting features, disease complications, and medications were collected. Chi-square and Fisher’s exact tests were used to assess for associations between medications and clinical characteristics.

**Results:**

Ninety-two children with idiopathic and 656 with juvenile idiopathic arthritis (JIA)-associated uveitis were identified. Indication (arthritis or uveitis) for medication use was not available for JIA patients; therefore, detailed analysis was limited to children with idiopathic uveitis. In this group, 94 % had received systemic steroids. Methotrexate (MTX) was used in 76 % of patients, with oral and subcutaneous forms given at similar rates. In multivariable analysis, non-Caucasians were more likely to be treated initially with subcutaneous MTX (*P* = 0.003). Of the 53 % of patients treated with a biologic DMARD, all received a tumor necrosis factor (TNF) inhibitor. TNF inhibitor use was associated with a higher frequency of cataracts (52 % vs 21 %; *P* = 0.001) and antinuclear antibody positivity (49 % vs 29 %; *P* = 0.04), although overall complication rates were not higher in these patients.

**Conclusion:**

Among idiopathic uveitis patients enrolled in the CARRAnet registry, MTX was the most commonly used DMARD, with subcutaneous and oral forms equally favored. Patients who received a TNF inhibitor were more likely to be ANA positive and have cataracts.

**Electronic supplementary material:**

The online version of this article (doi:10.1186/s12969-016-0069-5) contains supplementary material, which is available to authorized users.

## Background

Non-infectious juvenile uveitis is an inflammatory ocular disease that can lead to visual disability if not adequately controlled. Uveitis occurs as a primary disease (idiopathic uveitis) but can also be associated with systemic disease, typically juvenile idiopathic arthritis (JIA). In the pediatric population, uveitis remains a diagnostic challenge because children may not report symptoms until substantial visual damage has accrued. Rapid and effective control of the disease is essential because prolonged ocular inflammation is associated with higher rates of ocular complications and vision loss [1–3]. Unfortunately, there is no uniform approach to the evaluation and treatment of juvenile uveitis. In part, the lack of standardization is due to the limited number of clinical studies in this condition. Current treatment strategies for juvenile uveitis are mostly extrapolated from small, retrospective case series and there have been no randomized controlled trials in this patient population. In addition, few studies have examined treatment practice patterns in the care of juvenile uveitis, and these patterns may also vary between pediatric rheumatology and ophthalmology providers. Improved understanding of current practice may help inform future decisions regarding optimal care of these patients.

Glucocorticoids (oral and/or topical) are often the first step in treatment of anterior, non-infectious uveitis in children but long term use is discouraged due to side effects [4, 5]. Methotrexate is the most commonly prescribed steroid-sparing medication, and its therapeutic efficacy has been documented in several small case series [4, 6–10]. Methotrexate can be given either orally or subcutaneously. Subcutaneous administration has greater bioavailability compared to oral, which can be quite variable [11, 12]. However, for many patients, especially children, oral dosing is preferred. Other disease-modifying anti-rheumatic drugs (DMARDs) such as cyclosporine, tacrolimus, azathioprine, and mycophenolate mofetil are also used to treat uveitis, although most published reports have focused on adult, rather than pediatric, patients [13–23]. More recently, reports describe successful use of the TNF inhibitors infliximab and adalimumab in both JIA-associated and idiopathic uveitis [24–27]. In refractory cases of JIA associated uveitis, tociluzumab, abatacept, and rituximab have also been used [28–30].

Several groups have proposed guidelines for the treatment of JIA-associated uveitis based on literature review and expert opinion [31–33]. There is consensus among these treatment guidelines that topical steroids should be used first, followed by methotrexate for refractory disease. However, they differ in their recommendations on the use of systemic glucocorticoids, preferred route of methotrexate administration, and choice of second-line steroids sparing agents. Further, they have not yet been validated prospectively and it is unknown how closely providers adhere to these recommendations.

Comparative effectiveness research (CER) represents an alternative approach to the establishment of treatment guidelines by expert opinion. CER methodologies are well suited to study diseases such as juvenile uveitis that are relatively uncommon, making randomized controlled trials with large cohorts less feasible [34, 35]. Consensus Treatment Plans (CTPs), in which patients are treated according to one of several algorithms, are intended to help standardize care, while at the same time collecting information on outcomes that will allow comparison of different treatment practices. The implementation of CTPs and subsequent analysis of outcome data represent a form of CER. This approach has been trialed for multiple pediatric rheumatologic conditions, though not juvenile uveitis [36–39].

In this study, we examined data from CARRAnet, a large registry of North American pediatric rheumatology patients, in order to characterize current practice patterns in the treatment of juvenile uveitis. Better understanding of the factors influencing selection of steroid-sparing medications is necessary to inform the development of standardized treatment regimens and CTPs.

## Methods

### CARRAnet registry

CARRAnet was developed by the Childhood Arthritis and Rheumatology Research Alliance (CARRA) and is a multicenter registry of children with rare pediatric rheumatologic conditions (“CARRA registry” on https://clinicaltrials.gov). Over 50 pediatric rheumatology research centers in the United States and Canada have enrolled over 9000 children into the registry since its inception. Children were eligible to participate if they had 1 of 12 specified rheumatologic diseases that were being studied in the registry. The primary diagnosis at enrollment was determined by the child’s treating rheumatologist. The registry opened with recruitment of children with JIA in May 2010; recruitment of children idiopathic uveitis began in February 2012, at which time collection of detailed information on uveitis presentation and treatment was also introduced to the registry. Demographic and clinical data, including information on prior medication use and disease complications, were gathered by family interview, medical record review, and/or provider recall and subsequently entered into a secure online database. Consent/assent was obtained from all study participants.

### Uveitis data

After Institutional Review Board approval, we obtained de-identified enrollment data from children with JIA-associated and idiopathic uveitis who participated in the CARRA registry from June 2010 to September 2013. Although the registry was developed for prospective data collection, at the time of our inquiry, only data from a single, baseline registry enrollment visit was available for most patients. Data collected included uveitis type, number of affected eyes,duration of current episode (if active uveitis present), history of ocular complications, and current and prior medication use. Possible ocular complications listed included increased intraocular pressure, keratic precipitates, posterior synechiae, cataracts, macular edema, epiretinal membrane, snowbanks, snowballs, retinal vasculitis, macular atrophy, hypopyon, iris nodules, band keratopathy, and history of eye surgery. Information on topical therapies (steroid, mydriatic, and anti-glaucoma drops), systemic glucocorticoids, intra-ocular steroids, intravenous immunoglobulin, non-biologic disease-modifying antirheumatic drugs (DMARDs), and biologic agents were included in the registry. Long-term steroid use was defined as a history of systemic glucocorticoid treatment for > 1 month. Drug use was categorized as “current,” “past,” “never,” or “unknown.” For this analysis, “ever drug use” was defined as past and/or current use. Information on medication dose and dates of drug initiation/cessation were not included in the registry. Thus, while some longitudinal information regarding treatment could be inferred based on current and past use of medications, details such as duration of therapy were not available. For JIA patients, the indication for a systemic medication (arthritis vs. uveitis) was not recorded. Finally, data collection also included “ANA status,” where a positive response was defined as a history of testing positive for anti-nuclear antibody (ANA). Date of ANA testing was not included in the registry.

### Statistical analysis

Demographic differences between JIA-associated and idiopathic uveitis groups were compared with Mann-Whitney U and chi-square tests. Univariate analysis was performed between first-use of oral MTX and subcutaneous MTX, and between biologic and non-biologic groups, using the Mann-Whitney U-test for median age and Fisher’s exact test for percentages. Multivariable logistic regression (backward selection) was applied to identify predictive factors, with the likelihood ratio test used to assess significance of the independent predictors [40]. Odds ratios and 95 % confidence intervals (CI) were derived for significant multivariable predictors of route of MTX administration and for use of biologics.

In these univariate and multivariate analyses, Caucasian race included both Hispanic and non-Hispanic ethnicities. Due to the small number of subjects endorsing racial categories other than Caucasian/white and black/African American, racial categories were collapsed into Caucasian and non-Caucasian to allow sufficient patient numbers for statistical analysis. Statistical analysis was performed using IBM/SPSS Statistics (version 22.0, IBM, Armonk, NY). Two-tailed *P* < 0.05 were considered statistically significant.

## Results

### Patient characteristics

There were 92 children with idiopathic uveitis and 646 children with JIA uveitis identified in the CARRA registry (Table 1). Children with JIA uveitis were significantly younger at disease onset (median age 2.8 years) than children with idiopathic uveitis (median 8.5 years) (*P* < 0.001) (Table 1). Females were more frequently affected than males in both forms of the disease; however, patients with JIA uveitis were more likely to be female than those with idiopathic uveitis (78 % vs 55 %; *P* < 0.001) (Table 1). A greater percentage of patients with JIA uveitis (92 %) were Caucasian as compared to children with idiopathic uveitis (77 %) (*P* < 0.001) (Table 1).

**Table 1:** Subject characteristics

	Idiopathic Uveitis	JIA Uveitis
	*N* = 92	*N* = 646
Age at onset, yrs	8.5	2.8
Median (IQR)	(5.3-10.9)	(1.7-5.2)
Gender (n, %)		
Female	51 (55 %)	504 (78 %)
Male	41 (45 %)	142 (22 %)
Disease duration, yrs		
Median (IQR)	2.9 (1.2-5.2)	6.1 (3.2-9.5)
Race* (n, %)		
Caucasian	71 (77 %)	596 (92 %)
Black	12 (13 %)	22 (3 %)
Asian	4 (1 %)	16 (2 %)
Other	3 (3 %)	19 (3 %)
Unknown	2 (2 %)	0 (0 %)
Ethnicity (n, %)		
Hispanic	12 (13 %)	61 (9 %)
Non-Hispanic	79 (86 %)	580 (90 %)
Unknown	1 (1 %)	5 (0.5 %)
ANA (n, %)		
Positive	30 (32 %)	385 (60 %)
Negative	45 (49 %)	184 (28 %)
Unknown	17 (18 %)	77 (12 %)

Characteristics of the idiopathic and JIA uveitis patients are listed in the table. *Race was self-reported, and subjects could select more than one category

*JIA* juvenile idiopathic arthritis, *IQR* interquartile range

### Uveitis characteristics

**Fig. 1:**
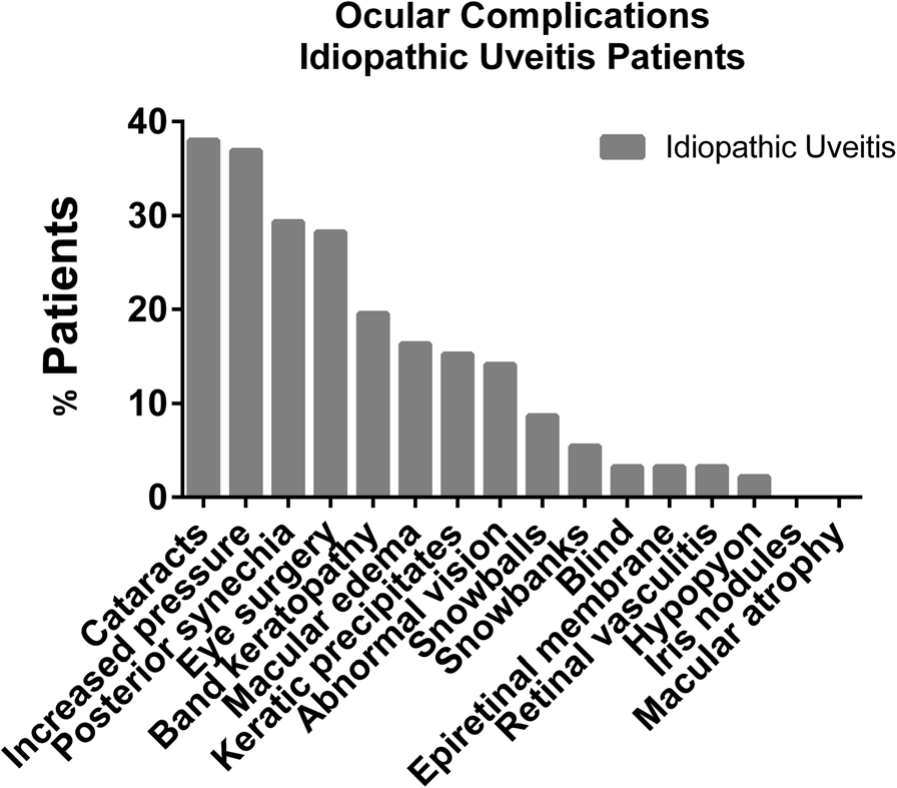
Ocular Complications in Idiopathic Uveitis. The number of idiopathic uveitis patients with each ocular complication is depicted in the figure

Information on uveitis disease characteristics and ocular complications was available for all patients with idiopathic uveitis but only 69 of 646 patients with JIA uveitis. Therefore, our evaluation of uveitis features was limited to patients with idiopathic uveitis. Anterior uveitis was the most common subtype of the disease (62 %), followed by panuveitis (21 %), intermediate uveitis (13 %), and posterior uveitis (4 %). Most children with idiopathic uveitis (77 %) had bilateral involvement. Ocular complications were common and noted in 71 (77 %) children with idiopathic uveitis: 35 had cataracts, 26 had undergone eye surgery, 13 had abnormal corrected vision, and 3 were blind in the affected eye (Fig. 1). Other frequently reported complications included posterior synechiae, band keratopathy, macular edema, and keratic precipitates. The majority of patients (64 children) reported having more than one complication. Among JIA uveitis patients for whom ocular complication data was known, cataracts again were the most common complication, although overall complication rate was lower (Additional file 1: Figure S1).

### Medication use

Data on medications ever used was available on all idiopathic and almost all (643/646) JIA uveitis patients (Tables 2 and 3). Use of DMARDs and biologic agents was similar in both groups. A majority of patients received a DMARD (83 % of idiopathic and 88 % of JIA uveitis patients). At least one biologic agent was used in 53 % of children with idiopathic uveitis and 56 % of children with JIA-associated uveitis. In patients with JIA uveitis, the indication for medication use was not specified and could have been due to either arthritis or uveitis disease activity. Therefore, in order to evaluate medication use for uveitis specifically, we restricted the remaining medication analysis to patients with idiopathic disease.

**Table 2:** DMARD use in idiopathic and JIA uveitis

Drug	Idiopathic Uveitis	JIA Uveitis
	*N* = 92 (%)	*N* = 643 (%)
Methotrexate	70 (76)	549 (85)
Oral	44 (48)	375 (58)
SQ	46 (50)	416 (65)
Mycophenolate mofetil	9 (10)	33 (5)
Cyclosporine	3 (3)	20 (3)
Sulfasalazine	1 (1)	47 (7)
Hydroxychloroquine	0 (0)	27 (4)
Leflunomide	0 (0)	20 (3)
Tacrolimus	0 (0)	8 (1)
Azathioprine	0 (0)	3 (0.4)
Cyclophosphamide	0 (0)	1 (0.2)

Data in this table represent “ever drug use” in patients with data available on DMARD drug exposure (92/92 patients with idiopathic uveitis and 643/646 patients with JIA uveitis)

*JIA* juvenile idiopathic arthritis, *SQ* subcutaneous

**Table 3:** Biologic agent use in idiopathic and JIA uveitis

Drug	Idiopathic Uveitis	JIA Uveitis
	*N* = 92 (%)	*N* = 643 (%)
TNF inhibitor	49 (53)	353 (55)
Adalimumab	18 (20)	196 (30)
Certolizumab	0 (0)	1 (0.2)
Etanercept	0 (0)	159 (25)
Golimumab	1 (1)	3 (0.5)
Infliximab	36 (39)	174 (27)
Abatacept	0 (0)	23 (4)
Anti-IL-1	0 (0)	9 (1.5)
Anakinra	0 (0)	7 (0.2)
Rilonacept	0 (0)	1 (0.2)
Canakinumab	0 (0)	1 (0.2)
Rituximab	0 (0)	4 (0.5)
Tocilizumab	0 (0)	4 (0.5)

Data in this table represent “ever drug use” in patients with data available on biologic drug exposure (92/92 patients with idiopathic uveitis and 643/646 patients with JIA uveitis)

*JIA* juvenile idiopathic arthritis

### Glucocorticoid use in idiopathic uveitis

Topical steroid drops were used in 90 % of idiopathic uveitis patients, with hourly dosing employed at some point in the disease course in 26 % (Table 4). Of the 5 children who did not receive topical steroid drops, 4 were treated with oral or IV glucocorticoids. None of the idiopathic uveitis patients received intra-ocular steroid injections. Almost all (94 %) of idiopathic uveitis patients had received systemic glucocorticoids. Further, 38 % of idiopathic uveitis patients had received long-term systemic glucocorticoids.

**Table 4:** Steroid use in idiopathic uveitis

	Idiopathic Uveitis N (%)
Topical steroid drops	
Yes	83 (90)
No	5 (5)
Unknown	4 (4)
Max topical steroid drop freq	
Hourly	24 (26)
12x/day	10 (11)
6x/day	4 (4)
4x/day	16 (17)
3x/day	6 (7)
2x/day	3 (3)
Daily	3 (3)
Unknown	21 (23)
Intraocular steroids	
Yes	0 (0)
No	85 (92)
Unknown	7 (8)
Systemic steroids	
Yes	86 (94)
No	4 (4)
Unknown	2 (2)
IV pulse steroids	
Yes	4 (4)
No	82 (89)
Unknown	6 (7)
Long-term systemic steroids	
Yes	35 (38)
No	50 (54)
Unknown	7 (8)

Topical and systemic steroid use in idiopathic uveitis patients is depicted in the table. *Max* maximum, *freq* frequency

### DMARD use in idiopathic uveitis

Of the 92 idiopathic uveitis patients, 70 (76 %) received methotrexate (MTX), making it the most frequently utilized steroid-sparing agent. Overall, the oral and subcutaneous (SQ) forms of MTX were used at similar rates (Table 2). In 96 % of patients who had received MTX, it was possible to determine the initial route of MTX administration prescribed to treat idiopathic uveitis. SQ MTX was used first in 54 % of children, while oral MTX was prescribed first in 46 %. A minority (29 %) of patients had been trialed on both formulations (SQ to oral in 10 patients; oral to SQ in 7 patients; in 3 subjects the order could not be determined). By univariate analysis, the initial use of the SQ MTX was associated only with non-Caucasian race (*P* = 0.046). This relationship persisted in the multivariable analysis with adjustment for age at disease onset, ethnicity, race, gender, uveitis type, and ocular complications (*P* = 0.003) (Table 5). DMARDs other than methotrexate that were less frequently used in idiopathic uveitis patients included cyclosporine (*N* = 3), mycophenolate mofetil (*N* = 9), and sulfasalazine (*N* = 1) (Table 2).

**Table 5:** Factors associated with initial route of MTX Administration in idiopathic uveitis

				Multivariable Logistic Regression		
Variable	Oral MTX	SQ MTX	Univariate *P* value	Odds Ratio	95 % CI	*P* value
	*N* = 31	*N* = 36				
Age at onset, yrs			0.06			0.61
Median (IQR)	9.4 (5.7-11.8)	7.1 (5.0-9.3)				
Gender			1.00			0.73
Female	13 (42 %)	15 (42 %)				
Male	18 (58 %)	21 (58 %)				
Ethnicity			1.00			0.72
Hispanic	2 (6 %)	2 (6 %)				
Non-Hispanic	29 (94 %)	34 (94 %)				
Race			0.046*	12.7	1.5-106.8	0.003*
Caucasian	27 (87 %)	22 (65 %)				
Non-Caucasian	4 (13 %)	12 (35 %)				
Uveitis type			0.65			0.51
Anterior	19 (61 %)	26 (72 %)				
Pan	4 (13 %)	5 (14 %)				
Intermediate	7 (23 %)	4 (11 %)				
Posterior	1 (3 %)	1 (3 %)				
ANA status			1.00			0.95
Positive	11 (44 %)	15 (44 %)				
Negative	14 (56 %)	19 (56 %)				
Complications**	25 (81 %)	28 (80 %)	1.00			0.73
Cataracts	15 (48 %)	16 (44 %)	0.81			0.94

Data in this table include subject characteristics associated with the route of methotrexate administration initially prescribed

*IQR* interquartile range, *SQ* subcutaneous, *CI* confidence interval, *ANA* antinuclear antibody

*Statistically significant, **Presence of at least one ocular complication

### Biologic use in idiopathic uveitis

Among the 49 idiopathic uveitis patients treated with a biologic medication, all received a tumor necrosis factor (TNF) inhibitor: infliximab (*n* = 36), adalimumab (*n* = 18), or golimumab (*n* = 1) (Table 3). Of these, all but 2 had also received methotrexate. In univariate analysis, patients who had received a TNF inhibitor were more likely to have a history of a cataract compared to patients who had never been exposed to a biologic agent (53 % vs. 21 %; *P* = 0.002) (Table 6). This association remained in multivariable analysis adjusting for age at disease onset, ethnicity, race, gender, uveitis type, ANA status, and ocular complications (odds ratio = 5.6; *P* = 0.001). In addition, multivariate modeling also revealed that a positive ANA, defined as a history of testing positive for ANA, was significantly associated with TNF inhibitor use after adjusting for other disease and patient characteristics (*P* = 0.04) (Table 6).

**Table 6:** Factors associated with biologic agent use in idiopathic uveitis

				Multivariable Logistic Regression		
Variable	Biologics	No Biologics	Univariate *P* value	Odds Ratio	95 % CI	*P* value
	*N* = 49	*N* = 43				
Age at onset, yrs			0.35			0.90
Median (IQR)	7.7 (5.1-10.2)	8.9 (6.8-11.4)				
Gender			0.21			0.07
Female	24 (49 %)	27 (63 %)				
Male	25 (51 %)	16 (37 %)				
Ethnicity			0.54			0.92
Hispanic	5 (10 %)	7 (16 %)				
Non-Hispanic	44 (90 %)	36 (84 %)				
Race			0.20			0.87
Caucasian	35 (73 %)	36 (86 %)				
Non-Caucasian	13 (27 %)	6 (14 %)				
Uveitis type			0.44			0.82
Anterior	27 (55 %)	30 (70 %)				
Pan	13 (27 %)	6 (14 %)				
Intermediate	7 (14 %)	5 (12 %)				
Posterior	2 (4 %)	2 (5 %)				
ANA status			0.07***			
Positive	20 (49 %)	10 (29 %)		3.0	1.0-8.6	0.04*
Negative	21 (51 %)	25 (71 %)		Reference		
Any complication	39 (81 %)	32 (74 %)	0.46			0.16
Cataracts	26 (53 %)	9 (21 %)	0.002*	5.6	1.8-17.2	0.001*

*IQR* interquartile range, *CI* confidence interval

*Statistically significant, ***Trend

### Multiple biologic agent use in idiopathic uveitis

Six idiopathic uveitis patients were treated with multiple biologic agents. All of these patients had received both infliximab and adalimumab. In 5 of these patients, the order of TNF inhibitor use could be determined: 3 patients switched from adalimumab to infliximab, while 2 patients switched from infliximab to adalimumab.

### Biologic use without preceding DMARD in idiopathic uveitis

Two patients included in the registry were treated with infliximab without a preceding DMARD. Neither of these patients had anterior uveitis, while both had ocular complications. The first patient was diagnosed with bilateral panuveitis and had developed snowballs and posterior synechiae. The second child had posterior uveitis complicated by retinal vasculitis and blindness in the affected eye.

## Discussion

Optimization of care for children with uveitis remains a challenging goal without better comparative effectiveness studies. These types of studies are particularly challenging to conduct in children given the rarity of uveitis and the practical considerations regarding therapeutic trials in children. In this study, we sought to present a preliminary overview of current practice patterns in the treatment of pediatric uveitis, using a large North American patient registry. This analysis includes description of one of the largest cohorts of pediatric idiopathic uveitis patients published to date. A total of 26 contributing centers were represented within this cohort.

We first examined steroid use in these patients. Somewhat surprisingly, nearly all patients had been treated with systemic glucocorticoids; indeed, more patients reported using systemic glucocorticoids than topical steroids. About a third of patients reported long-term use of systemic glucocorticoids, and several patients had received intravenous glucocorticoid pulses. In contrast to the pattern of steroid use documented in our cohort of patients, published treatment guidelines for JIA-associated uveitis reserve systemic glucocorticoids for patients with severe ocular inflammation (grades 3+ or 4+) or impending vision loss [31, 32]. The discrepancy between systemic glucocorticoid use in our cohort compared to the treatment guidelines may represent a point where current treatment practices deviate from expert opinion. It is possible that this deviation is due to differences in treatment approaches between idiopathic and JIA-associated uveitis. Alternatively, the high rate of systemic glucocorticoiduse in our cohort could be explained by higher disease severity, a possibility that is difficult to assess as the CARRAnet registry did not include information about grade of inflammation. However, the overall rate of ocular complications in our cohort (80 %) is within the range of prior reports in juvenile uveitis [1, 2, 41].

The use of steroid-sparing medications in idiopathic uveitis was then evaluated. Most patients had received methotrexate, in either oral or subcutaneous form. Subcutaneous methotrexate has higher bioavailability than oral dosing, but use of subcutaneous methotrexate for initial therapy is not clearly standard among providers. We found that idiopathic uveitis patients in our study were evenly divided between oral and subcutaneous dosing. Univariate and multivariate analysis demonstrated that subcutaneous administration was used more frequently for non-Caucasians than Caucasians. A possible explanation could be that non-Caucasians had, or were at least perceived to have, more severe disease. However, other characteristics such as complication rate, age of onset, gender, and ANA status were not different between patients who were treated with oral versus subcutaneous methotrexate. There is limited literature on the role of race and ethnicity in juvenile uveitis. In one study of JIA-associated uveitis, African-American children tended to have worse physician global assessment scores [42]. Another study has also suggested that African-American patients may have more severe disease [43]. In contrast, white race was a predictor of remission in one study of JIA-associated uveitis, but race was not a factor affecting development of complications [44]. Whether these findings are applicable to idiopathic uveitis patients is less clear. Other factors that may influence medication selection were not able to be captured in our study; these include patient preference, concerns about medication compliance, and regional availability of injectable methotrexate due to drug shortages [45].

More than half of our idiopathic uveitis cohort received biologic therapy, all in the form of TNF inhibitors. Infliximab was used twice as frequently as adalimumab, which was interesting given that recent literature seems to have favored adalimumab over infliximab in some analyses [46, 47]. One explanation for this may be the earlier availability of infliximab. However, our patients were first enrolled beginning in 2010; adalimumab use in juvenile uveitis was reported as early as 2006 [48]. There were no racial differences between patients who had or had not received TNF inhibitor therapy. However, patients treated with TNF inhibitors were more likely to be ANA positive and to have cataracts, again suggesting that these patients either had, or were perceived to have, more severe disease. Unfortunately, the design of the registry did not allow the ability to capture whether ANA positivity and cataract development preceded or followed TNF inhibitor administration. Several studies have identified ANA positivity as a risk factor for ocular complications and visual acuity loss in JIA-associated uveitis; however, other studies have not replicated this finding [2, 49–51]. There is, thus, no clear explanation of the association between ANA positivity and TNF inhibitor use in our cohort apart from the possible perception of increased disease severity.

Cataract development may be secondary to disease activity or chronic steroid use. In our cohort, patients with cataracts had higher frequencies of other ocular complications but similar rates of glucocorticoiduse compared to children without cataracts. Thus, differences in disease severity more likely explain the association between cataract development and the use of TNF inhibitors. Further understanding of this relationship is limited by the available data, which in this registry database did not specify the timing of TNF inhibitor initiation with regard to cataract formation.

### Limitations

Although the size of our idiopathic uveitis pediatric cohort was relatively large, interpretation of the impact of specific medications on patient outcome is limited by the lack of follow up. This was a relatively early cross-sectional look at enrollment data, but collection of data from follow up visits remains ongoing. We hope that over time, further analyses will allow more detailed examination of the factors that influence the selection of medications, as well as their efficacy.

The CARRAnet database did not include data on the indication for medication use in JIA uveitis patients. Therefore, it was not possible to determine if a medication was used for active arthritis and/or uveitis. Thus, we limited the majority of our analysis to idiopathic uveitis patients. It is unclear if our findings are applicable to JIA uveitis patients, given the differences in race, gender, and ANA status in both groups which may be associated with differences in disease severity, and subsequently medication use. The database also did not collect information on medication dosage, which can also vary greatly from provider to provider and also influence the decision for whether to move on to another agent. Future iterations of this database will hopefully allow more granular analysis of medication dosage.

Finally, our cohort may be influenced by selection bias, as these patients were all under the care of a pediatric rheumatologist. Patients with milder disease may be managed solely by ophthalmologists without coming to the attention of a rheumatology provider.

## Conclusions

There is not yet a commonly accepted, standardized approach in the treatment of juvenile idiopathic uveitis. Analysis of a North American pediatric rheumatology data registry identified points of consensus and divergence in the current treatment practices for this disease. Compared to expert treatment guidelines, clinicians used systemic steroids more frequently and for prolonged courses. Steroid-sparing treatment strategies were generally similar for children with JIA-associated and idiopathic uveitis. Among patients with idiopathic uveitis, almost all were treated with methotrexate, although oral and subcutaneous forms of the medication were equally favored. Non-Caucasian patients were more likely to be prescribed subcutaneous rather than oral methotrexate. More than half of our idiopathic uveitis patients were also treated with a TNF inhibitor, and infliximab was used twice as often as adalimumab. TNF inhibitor use may reflect higher disease severity, or the perception of higher disease activity, as it was also associated with higher rates of ANA positivity and cataract formation.

Our findings on the treatment of juvenile uveitis in North America lay the foundation for future investigation into identified areas of treatment variability. Comparative effectiveness research, particularly CTPs, will be helpful in studying systemic steroid use and the efficiency of oral versus subcutaneous methotrexate, and the TNF inhibitors infliximab and adalimumab.

## Additional file

Additional file 1: Figure S1. Ocular complications in JIA associated uveitis. (TIF 294 kb)

## Abbreviations

CARRA: Childhood Arthritis and Rheumatology Research Alliance
CER: comparative effectiveness research
CTP: consensus treatment plans
DMARD: disease-modifying anti-rheumatic drug
JIA: Juvenile idiopathic arthritis
MTX: methotrexate
SQ: subcutaneous
TNF: tumor necrosis factor
